# War-related trauma and post-traumatic stress disorder prevalence among Syrian university students

**DOI:** 10.1192/bjo.2021.163

**Published:** 2021-06-18

**Authors:** Fatema Mohsen, Yousef Latifa, Bisher Sawaf

**Affiliations:** 1 Syrian Private University; 2 Damascus University; 3 Hamad medical corporation

## Abstract

**Aims:**

PTSD is one of the most prevalent mental disorders in war-affected regions. Syria has endured 10 years of war and yet little is known about the impact of the conflict on the well-being of Syrians who remain. This study aimed to provide an estimated prevalence of PTSD among trauma-exposed university students in Deir-ez-Zor, Syria, a war-ridden region, that was under siege by the Islamic State of Iraq and the Levant (ISIS) for over 3 years. Moreover, we aimed to study the different types of trauma to which the students were exposed and studied the association between PTSD and multiple covariates including, socio-demographic characteristics, smoking habits, academic performance, and stress levels, and identify factors that influence the development of PTSD symptoms.

**Method:**

A descriptive cross-sectional study design was used on a sample of Al-Furat university students in Deir-ez-Zor. We collected data on socio-demographics, trauma exposure, and stress levels. PTSD Checklist for DSM-5 was used to carry out PTSD diagnosis and to determine the severity of the disorder.

**Result:**

A total of 833 Syrian students were recruited into the study, the mean was 22.4 ± 3.2 years. Of those, (22.2%) have been displaced 3 times, while (18.8%) were displaced over 5 times. (86.4%) reported experiencing at least one traumatic event, (33.8%) of the participants were exposed to one traumatic event, and (44.7%) experienced four or more traumatic events. PTSD prevalence was (28.2%), and the highest PTSD rates were found among students who were forced into sexual acts (46.3%), followed by those who witnessed childhood trauma or violence and those who witnessed violence as adults (42.6%). Sample distribution over stress levels was as follows: normal (39.5%), mild (16.0%), moderate (17.8%), severe (17.3%), and extremely severe (9.8%). A statistically significant association was found between PTSD prevalence and stress severity (p = 0.000). A significant association was found between PTSD and internal displacement (p = 0.032), academic year (p = 0.002), and social-economic status (p = 0.000). Binary logistic regression revealed that smokers (vs non-smokers, OR = 0.259, p = .034) and third-year students (vs fifth year, OR = 0.44, p = .019) were significantly associated with PTSD.

**Conclusion:**

The results presented in this research revealed a high prevalence of trauma exposure and PTSD among a sample of university students in Deir-ez-Zor. These findings call for immediate actions to help the affected population in restoring their mental health, so they can be prepared to face the challenges and demands of the post-conflict period.

